# Effects of Lycopene Supplementation on Bone Tissue: A Systematic Review of Clinical and Preclinical Evidence

**DOI:** 10.3390/ph18081172

**Published:** 2025-08-08

**Authors:** Arles Naisa Amaral Silva, Gabriel Pereira Nunes, Danilo Vinicius Aparecido de Paula Domingues, Priscila Toninatto Alves De Toledo, Olumide Stephen Akinsomisoye, Rinaldo Florencio-Silva, Paulo Sérgio Cerri

**Affiliations:** 1 Department of Morphology, Genetics, Orthodontics and Pediatric Dentistry, School of Dentistry, São Paulo State University—UNESP, Araraquara, SP 14801-903, Brazil; arles.naisa@unesp.br (A.N.A.S.); oakin@oauife.edu.ng (O.S.A.); 2 Department of Prosthodontic and Periodontology, School of Dentistry, São Paulo State University of Campinas—UNICAMP, Piracicaba, SP 13414-903, Brazil; gabriel.p.nunes@unesp.br; 3 Department of Periodontology, Dental Research Division, Guarulhos University—UNG, Guarulhos, SP 07023-070, Brazil; dandanvinicius@gmail.com; 4 Department of Operative Dentistry, Endodontics and Dental Materials, Bauru School of Dentistry, University of São Paulo—USP, Bauru, SP 17012-901, Brazil; priscilatoninatto@hotmail.com; 5 Department of Physiological Sciences, Obafemi Awolowo University, Ile Ife 220005, Nigeria; 6 Department of Morphology and Genetics, Division of Histology and Structural Biology, Paulista School of Medicine—EPM, Federal University of São Paulo—UNIFESP, São Paulo, SP 04023-062, Brazil; silva05@unifesp.br

**Keywords:** lycopene, bone tissue, bone remodeling, bone loss, bone resorption, systematic review

## Abstract

**Background**: Bone tissue undergoes continuous remodeling, and imbalances in this process can lead to osteometabolic disorders, such as osteoporosis. Thus, bioactive compounds, like lycopene (LYC), have been investigated for their potential protective effects in bone health. This systematic review (SR) aimed to evaluate the effects of LYC supplementation on bone tissue. **Methods**: The SR was registered in PROSPERO (CRD42023417346) and followed the PRISMA guidelines. A comprehensive search was conducted in electronic databases up to May 2025. Two independent reviewers selected clinical trials and animal studies evaluating the effects of LYC supplementation in bone tissue. Methodological quality and risk of bias were assessed using the Cochrane Risk of Bias tool for randomized controlled trials, the Newcastle–Ottawa Scale for non-randomized clinical studies, and the SYRCLE tool for animal studies. **Results**: A total of 21 studies met the eligibility criteria, consisting of 6 clinical trials and 15 studies in animal models. LYC supplementation promotes an increase in bone mineral density, preserves trabecular microarchitecture, stimulates osteoblastic activity, and inhibits osteoblast apoptosis. **Conclusions**: LYC supplementation promotes beneficial effects on both the formation and preservation of bone tissue, suggesting that this carotenoid may represent a potential adjuvant strategy in the management of osteometabolic disorders.

## 1. Introduction

Bone remodeling is an essential physiological mechanism for maintaining skeletal integrity through the resorption of old bone tissue and formation of bone matrix [1,2,3]. This process is tightly regulated by coordinated functions of osteoblasts, osteocytes, and osteoclasts [2,3]. However, it can be disrupted by various factors, including hormonal changes, particularly those related to menopause, age-related factors, physical activity levels, medications, and underlying health conditions [4].

In young/adult healthy individuals, bone remodeling maintains a balanced state between bone resorption by osteoclasts and bone formation by osteoblasts, enabling the skeleton to renew and adapt to physical and metabolic demands [3]. As individuals age, however, this balance can be disrupted, whereby bone resorption exceeds bone formation, thus leading to bone loss. Menopause is especially critical in this regard, as declining estrogen levels lead to increased bone resorption and accelerated bone loss [5]. The reduction in estrogen levels is a major contributor to osteoporosis, a condition characterized by decreased bone mass and deterioration of bone microarchitecture, leading to an increased risk of fractures [5,6]. It has been estimated that around 30% of postmenopausal women develop osteoporosis, which reinforces the importance of early diagnosis and intervention to maintain bone health [7]. There are several strategies for the prevention and treatment of osteoporosis, including calcium and vitamin D supplementation, regular physical exercise, and pharmacological approaches designed to strengthen bones and reduce fracture risk [8,9,10].

Bone disorders, such as osteoporosis, remain a significant public health concern, particularly among aging individuals and postmenopausal women [7,9]. While pharmacological interventions, such as bisphosphonates, selective estrogen receptor modulators, and anabolic agents, can effectively reduce fracture risk and help maintain bone mass, their use is often limited by adverse effects, poor long-term adherence, and high financial costs [6,8]. These limitations have spurred growing interest in complementary approaches, especially in the use of natural compounds with antioxidant, anti-inflammatory, and bone-regulating properties [11,12,13]. Notably, antioxidants, phytoestrogens, natural anti-inflammatory agents, and plant-derived bioactives have shown promise due to their ability to act on various biological pathways involved in bone homeostasis [13]. FDA-approved drugs, including both antiresorptive and anabolic agents, though effective, may still lead to complications, such as atypical fractures, and are associated with high treatment costs [6,11]. Consequently, natural alternatives that offer fewer side effects have gained attention [12]. Natural pharmacology is emerging as a safer and more versatile therapeutic approach, with potential for synergistic action, reduced resistance risk, and minimal environmental impact [12]. Moreover, it aligns with traditional medical practices and supports sustainability principles [13]. A primary target of these therapies is oxidative stress, since reactive oxygen species (ROS) disrupt bone metabolism and contribute to bone loss [14,15], particularly in postmenopausal women [16,17]. In this context, antioxidants, especially those abundant in carotenoid-rich foods, are being investigated for their ability to counteract ROS-induced damage and promote bone health [18,19]. Beyond their bone-protective effects, these compounds may also provide broader systemic benefits [20], making them promising candidates for long term osteoporosis management.

Lycopene (LYC), a lipid-soluble carotenoid predominantly found in tomatoes and other red fruits, is widely recognized for its potent antioxidant properties [21]. This compound has garnered increasing attention from researchers and clinicians due to its potential therapeutic benefits in various health conditions, including cancers [22,23], cardiovascular diseases [24], aging [25], obesity, and diabetes mellitus [26]. Notably, LYC has also been implicated in promoting skeletal health [27], with evidence suggesting that it improves bone quality and attenuates bone loss in obese and osteoporotic animal models [28,29]. The antioxidant properties of LYC help to reduce oxidative stress, which is a significant factor in bone breakdown, especially in certain conditions, like osteoporosis. By neutralizing ROS, LYC may help protect osteoblasts, the cells responsible for bone formation, and reduce the activity of osteoclasts, which are involved in bone resorption [30,31].

Moreover, LYC has been reported to enhance the expression of genes involved in bone formation and inhibit those associated with bone resorption, playing an important role in the maintenance of bone tissue [28,32]. These effects make LYC a promising candidate for not only preventing bone loss but also improving overall bone strength, particularly in populations at risk of osteoporosis, such as postmenopausal women and individuals with metabolic disorders. Despite these promising findings regarding dietary antioxidants and their potential effect on bone metabolism, there is a lack of comprehensive syntheses specifically focused on LYC and overall bone health. A previous review has examined general effects of antioxidants on bone health [14], but none have systematically evaluated LYC across both preclinical and clinical studies. Therefore, the aim of the current systematic review was to critically assess the effect of LYC supplementation on bone health outcomes in either preclinical or clinical studies.

## 2. Results

### 2.1. Study Selection

**Figure 1:**
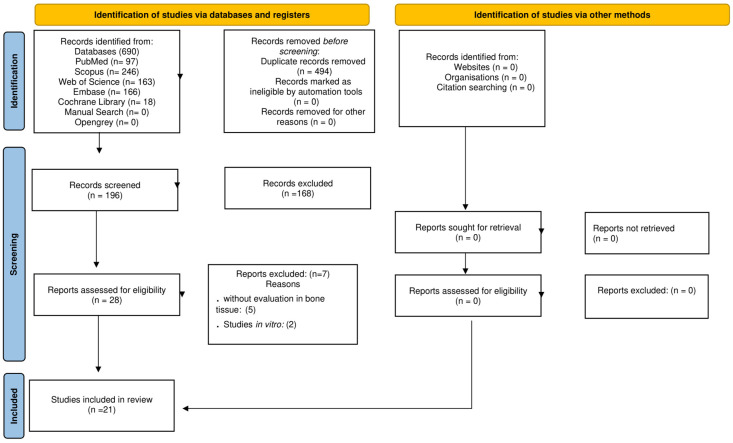
PRISMA 2020 flow diagram illustrating the number of studies identified, screened, assessed for eligibility, and included in the review.

The study selection process is summarized in Figure 1. An initial search of the databases identified a total of 690 studies, distributed as follows: there were 97 from PubMed/MEDLINE, 246 from Scopus, 166 from Embase, 163 from Web of Science, and 18 from the Cochrane Library. After removing 494 duplicates, 196 unique records remained for the screening phase. Title and abstract screening reduced this number to 28 articles, which were selected for full-text evaluation. Following a comprehensive assessment, 7 studies were excluded, resulting in 21 studies that met the eligibility criteria and were included in this systematic review. These consisted of 6 clinical trials [11,33,34,35,36,37] and 15 preclinical studies in animal models [17,18,28,29,30,32,38,39,40,41,42,43,44,45,46]. Inter-rater reliability for article inclusion was excellent, with a Cohen’s kappa coefficient (k) of 0.94 across all databases.

### 2.2. Characteristics of Studies

The eligible studies, published between 2007 and 2024, were conducted in a range of countries, reflecting broad geographic representation. These countries included China [28,29,38,41,43,45,46], Canada [34,35,36,37], Brazil [30,32,39], Italy [11,40], Japan [18,44], India [33], Egypt [42], and Saudi Arabia [17].

#### 2.2.1. Clinical Trials

Among the six eligible clinical studies, two were randomized controlled trials [33,35], while the remaining four were non-randomized clinical studies [11,34,36,37]. Collectively, these studies included 409 postmenopausal women, with ages ranging from 54 to 69 years. The administered doses of LYC varied across studies, ranging from 8 mg/day to 150 mg/day. LYC was delivered through different formulations, including capsules [33,35], tomato juice [35], and mature tomato juice [11]. LYC was typically administered once or twice daily.

All studies measured LYC concentrations using high-performance liquid chromatography (HPLC) [11,33,34,35,36,37]. Serum LYC levels were analyzed in five studies [33,34,35,36,37], whereas one study evaluated the LYC content of tomato sauce [11]. To quantify total lipid profiles, C-reactive protein (CRP) levels [33], and markers of bone turnover and formation [34,35,36,37], enzyme-linked immunosorbent assay (ELISA) kits were used. In contrast, one study employed the chemiluminescent immunoassay method for the evaluation of biochemical parameters, including CRP and transaminases [11]. Additionally, three studies used the Trolox-equivalent antioxidant capacity (TEAC) assay to assess antioxidant status [34,35,36]. The duration of follow-up among participants ranged from one to six months across studies (Table 1).

#### 2.2.2. Preclinical Models

Fifteen studies using animal models were included, as detailed in Table 2. A total of 797 animals were analyzed, with ages ranging from 6 to 20 weeks and body weights between 150 g and 300 g. The studies employed various experimental models, including ovariectomy-induced osteoporosis [17,18,28,30,32,39,43,45,46], streptozotocin-induced diabetes [41], high-fat diet-induced obesity [29], glucocorticoid-induced osteoporosis [40,42], growth-phase rats [44], and senescence using SAMP6 (Senescence-Accelerated Mouse Prone 6) mice [38]. The animal strains used included Sprague–Dawley [18,28,40,41,43,44,46], Wistar [17,30,32,39,45], and SAMP6 and SAMR1 [38]. Two studies did not mention the animal strain used [29,42].

LYC was administered for periods ranging from 4 to 16 weeks, predominantly via intragastric gavage administered daily [17,28,29,30,32,38,39,40,41,42,43,45,46]. In contrast, two studies incorporated LYC into the animals’ diet [18,44]. The compound was dissolved in various vehicles, including corn oil [17,38,41,43,45], sunflower oil [28,29], cottonseed oil [18,44] and water [30,32,39,42,46]. One study did not specify the vehicle used for LYC administration [40].

LYC doses varied from 10 to 100 mg/kg/day. A dose of 10 mg/kg/day was reported in four studies [30,32,40,46], while 15 mg/kg/day was administered in another four [17,28,29,46]. Higher doses included 20 mg/kg/day [45,46], and 30 to 50 mg/kg/day in several studies [17,28,38,39,41,42,43]. The highest reported dose was 100 mg/kg/day [41]. Additionally, two studies administered LYC in concentrations expressed in parts per million (ppm), ranging from 50 to 200 ppm [18,44].

A variety of methodologies were employed to assess outcomes. The most utilized technique was micro-computed tomography (μ-CT) to evaluate bone microarchitecture [17,28,29,30,38,40,41,43]. Biomechanical assessments of bone strength included three-point bending tests [17,28,29,38,41,45], compression testing [17], push-out testing [43], and mechanical rupture testing [18,44]. Serum biomarkers of bone metabolism were analyzed using ELISA kits [28,38,41,45], automated biochemical analyzers [45], and the modified Lowry method [18,44]. Gene expression related to bone formation and remodeling was evaluated by real-time quantitative reverse transcription polymerase chain reaction (qRT-PCR) in several studies [17,30,32,38,40].

### 2.3. Outcomes Related to LYC Supplementation

#### 2.3.1. Clinical Trials

All eligible clinical trials evaluating LYC administration in postmenopausal populations reported significant improvements in clinical outcomes, biochemical parameters, and bone metabolism markers [11,33,34,35,36,37]. LYC supplementation preserved bone mineral density (BMD), and increased serum LYC concentrations and P1NP levels, while only modest reductions in β-C terminal telopeptide (β-CTX) concentrations were observed [33]. Comparative analyses demonstrated greater osteoprotective effects in LYC-supplemented groups, characterized by lower bone alkaline phosphatase activity and prevention of BMD decline compared to non-supplemented controls [11].

A one-month dietary restriction of LYC in postmenopausal women led to significant changes in biomarkers of oxidative stress and bone resorption. The exclusion of LYC-rich foods led to a significant decrease in serum carotenoid levels, particularly LYC, accompanied by an increase in oxidative stress markers [34]. Notably, there was a significant rise in N-telopeptide (NTx), a marker of bone resorption, and in the activity of the antioxidant enzyme glutathione peroxidase (GPx), whereas the activities of catalase (CAT) and superoxide dismutase (SOD) were reduced [34]. Supporting these findings, MacKinnon et al. (2011) demonstrated positive effects of LYC supplementation over time, showing a significant increase in serum LYC levels following the administration of juice or capsules containing Lyc-O-Mato^®^ tomato extract. This increase was accompanied by a reduction in NTx levels in postmenopausal women. Based on these results, the authors suggested that LYC intervention, in the form of capsules or juice at a dosage of 30 mg/day, may contribute to reducing the risk of osteoporosis [35].

LYC supplementation has been shown to effectively reduce bone resorption in postmenopausal women, highlighting its potential as a viable dietary intervention for preserving bone health [36,37]. Clinical studies have shown an inverse correlation between elevated serum LYC levels and reduced N-telopeptide (NTx) concentrations [36,37], suggesting a reduction in osteoclastic activity. Furthermore, biochemical analyses revealed that higher circulating LYC levels were associated with decreased protein oxidation markers [37], pointing to a dual mechanism of action that involves both anti-resorptive and antioxidant pathways.

#### 2.3.2. Preclinical Studies

The 15 studies conducted in animal models consistently demonstrated that LYC supplementation has beneficial effects on bone metabolism [11,17,18,28,29,30,32,38,39,40,41,42,43,44,45,46]. In ovariectomized (OVX) rat models, a well-established model for studying postmenopausal osteoporosis, LYC treatment reduced femoral epiphyseal bone loss, enhanced osteoblast activity, and preserved trabecular bone structure [32]. Similarly, Semeghini et al. (2022) reported that LYC administration significantly increased the total number of osteoblasts and osteocytes, while reducing both the volume and number of osteoclasts, indicating a potential anabolic and anti-resorptive effect of LYC on bone tissue under estrogen-deficient conditions [30].

Moreover, LYC administration has been shown to attenuate bone loss in OVX rat models by promoting osteogenesis and inhibiting adipogenesis, primarily through modulation of the FoxO1/PPARγ signaling pathway under oxidative stress conditions [28]. In obese murine models, LYC intake correlated with lower body weight gain, improved glycemic and lipid profiles, and preservation of biomechanical bone strength and microarchitecture [29]. In OVX-induced osteoporotic rats, administration of 45 mg/kg LYC was linked to greater bone neoformation compared to untreated groups [39]. In addition, LYC-treated animals showed higher BMD, BMD/weight ratio, bone mineral content (BMC), and BMC/weight ratio, along with reduced serum calcium, phosphorus, interleukin-6, osteocalcin [45], and alkaline phosphatase levels [45,46].

In senile osteoporosis models using SAMP6 mice [38], LYC supplementation was associated with reduced oxidative stress, lower cellular senescence, and decreased secretion of senescence-associated secretory phenotype (SASP) inflammatory factors. Osteogenic activity also appeared enhanced, with greater bone formation observed [38]. In diabetic osteoporosis rat models [41], LYC intake was linked to increased osteoprotegerin (OPG) expression, decreased RANKL expression, and reduction in the number of osteoclasts and in the number of adipocytes in the bone marrow, contributing to the maintenance of bone tissue structure [41].

Under osteopenic conditions, LYC supplementation has been linked to improved osseointegration of titanium implants [43]. Push-out testing revealed a 69.3% increase in mechanical strength compared to untreated OVX controls, reflecting enhanced implant fixation [43]. Micro-CT analyses indicated greater bone volume, increased trabecular thickness, and reduced trabecular spacing in LYC-treated groups [43]. In parallel, Iimura et al. (2014, 2015) reported significant increase in BMD in both growing and OVX rats [18,44], accompanied by elevated bone alkaline phosphatase activity and reduced urinary deoxypyridinoline levels [17,44].

In models of glucocorticoid-induced osteoporosis [40,42], LYC use was associated with increased BMD, preservation of trabecular architecture, reduced osteoclastic activity, and decreased osteoblast apoptosis, highlighting its protective role against bone resorption.

### 2.4. Risk of Bias Within Studies

**Figure 2:**
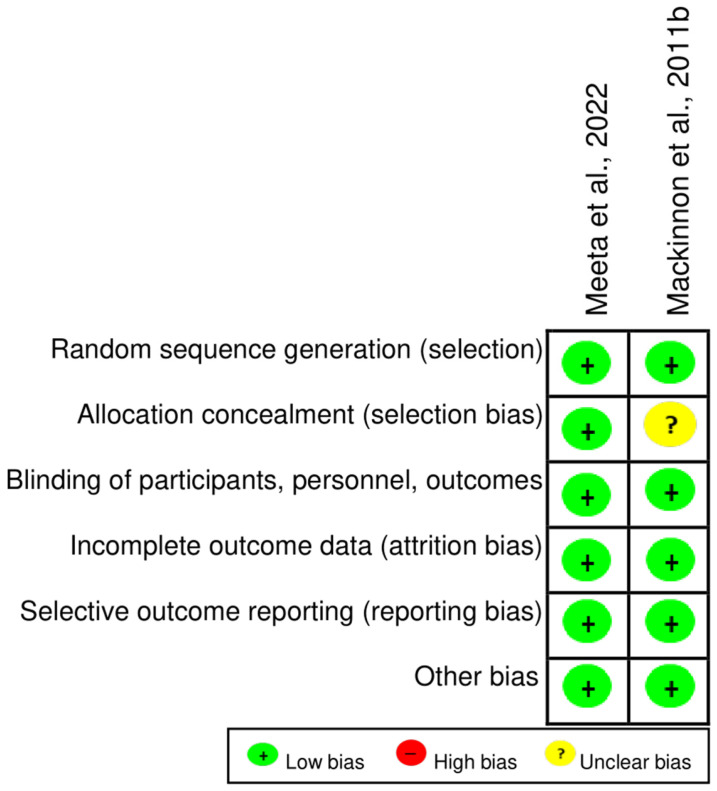
Summary of risk of bias assessment based on the Cochrane tool. Meeta et al., 2022 [33]; Mackinnon et al., 2011 [34].

**Figure 3:**
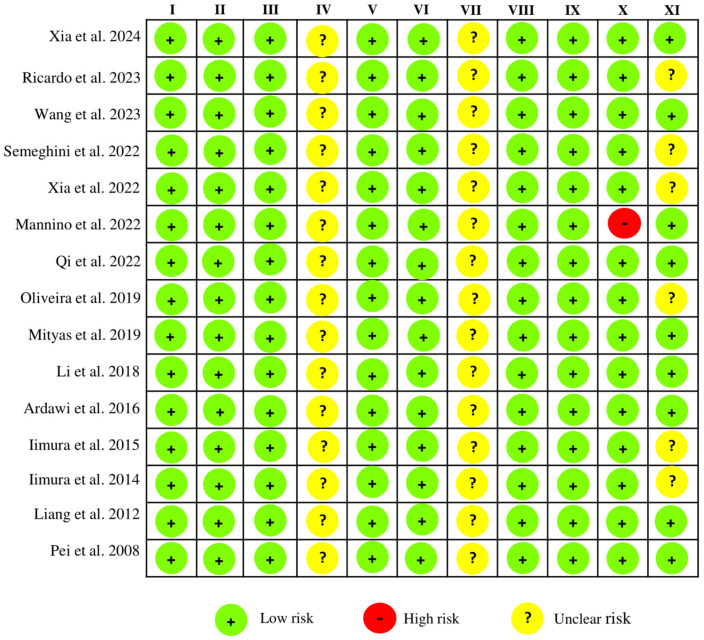
Risk of bias assessment of the selected animal studies using the SYRCLE’s Risk of Bias tool. Domains: I—sequence generation; II—baseline characteristics; III—allocation concealment; IV—random housing; V—performance blinding; VI—random outcome assessment; VII—blinding of outcome assessment; VIII—incomplete outcome data; IX—selective outcome reporting; X—other sources of bias; XI—sample size justification. Xia et al., 2024 [28]; Ricardo et al., 2023 [39]; Wang et al., 2023 [38]; Semeghini et al., 2022 [30]; Xia et al., 2022 [29]; Mannino et al., 2022 [40]; Qi et al., 2022 [41]; Oliveira et al., 2019 [32]; Mityas et al., 2019 [42]; Li et al., 2018 [43]; Ardawi et al., 2016 [17]; Iimura et al., 2015 [18]; Iimura et al., 2014 [44]; Liang et al., 2012 [45]; Pei et al., 2008 [46].

The risk of bias was assessed according to study design, namely RCTs (Figure 2), non-randomized clinical studies (Table 3), and animal models (Figure 3). Based on the Cochrane Collaboration’s tool for risk of bias assessment, both RCTs generally showed a low risk of bias [33,35]. However, one study [35] received an “unclear” rating for the domain of allocation concealment due to insufficient methodological details (Figure 2). According to the NOS qualifier, the non-randomized clinical studies showed a low risk of bias (Table 3). The domains that were not awarded a star included “Additional Factors” due to the lack of clear information on approaches related to external factors that could influence the analysis [34,36,37], and “Inadequate Follow-up”, attributed to the short follow-up period of just one week, which limits the evaluation of long-term effects [37].

The SYRCLE risk of bias tool was used to assess the methodological quality of animal studies. Overall, the studies exhibited a low risk of bias, although some domains were not clearly reported (Figure 3). None of the studies offered adequate details regarding animal handling in the “Random Housing” domain or provided clear information on blinding for outcome assessment. Under “Other Sources of Bias”, Mannino et al. (2022) disclosed funding from the pharmaceutical industry [40]. In addition, the studies did not provide information to justify the sample size [18,29,30,32,39,44]. In general, the remaining domains were classified as having a low risk of bias.

## 3. Discussion

LYC, a potent carotenoid antioxidant abundantly found in tomatoes and other red-hued fruits, has gained recognition as a promising nutraceutical for bone health [21,47]. To the best of the authors’ knowledge, this is the first systematic review to assess the effects of LYC supplementation specifically on bone tissue. This review critically evaluated the research carried out on the effects of LYC in bone tissue, focusing on methodological approaches, key findings, underlying mechanisms, and clinical implications. All eligible clinical trials evaluating LYC administration in postmenopausal populations have shown significant improvements in clinical outcomes, biochemical parameters, and markers of bone metabolism [11,33,48]. Similar findings were observed in animal models. LYC administration attenuated bone loss in ovariectomized rats [28], improved glycemic and lipid profiles [29], and reduced serum calcium, phosphorus, interleukin-6, osteocalcin, and alkaline phosphatase levels [45].

One of the key major activities of LYC is its osteoprotective effect. In six eligible clinical studies reported in this review, postmenopausal women treated with LYC presented an increase in serum LYC concentrations and type I procollagen N-terminal propeptide (P1NP) levels and had preserved BMD [33]. The alkaline phosphatase activity in bone was lower and better prevented a decline in BMD than in non-supplemented controls [11]. The promising findings evidenced in this systematic review are attributed to the biological properties of LYC, which may positively influence bone functionality and homeostasis [17,48]. LYC enhances the function of the osteoblast-like cell Saos-2 through various mechanisms, such as activating the ERK 1/2 and WNT/β-catenin cellular pathways, as well as upregulating RUNX2 and COL1A1 mRNA levels, while downregulating RANKL/RANK [11]. The primary mechanisms by which LYC carries out its bone-protective effects include its ability to neutralize ROS and suppress pro-inflammatory cytokines. In addition, oxidative stress accelerates bone loss by stimulating osteoclast activity and inhibiting osteoblast function [16]. These events reduce bone mass and bone turnover [32].

Meanwhile, the OPG/RANKL/RANK axis is critical in osteoclastogenesis [49,50,51]. Oxidative stress upregulates RANKL/RANK and downregulates OPG via ERK1/2, JNK, and other transcription factors [52]. RANKL/RANK enhances osteoclast differentiation and function via preosteoclast receptors, promoting osteoclast formation and bone resorption [52,53]. However, the potent antioxidant capacity of LYC plays a role in maintaining redox balance in bone tissue [11]. Cytokines released by immune cells and residents in inflamed tissues induce the formation and activity of osteoclasts, leading to increased bone resorption [54,55]. However, LYC reduces the levels of TNF-α, IL-6, and other inflammatory mediators that stimulate osteoclastogenesis, mitigating the deleterious effects on bone tissue [56,57]. The beneficial effects of LYC in bone tissue have been reported in both in vitro and in vivo studies. These studies have shown that LYC inhibits osteoclast differentiation by downregulating RANKL/NF-κB signaling pathways [11,41]. Additionally, LYC induces osteoblast function by enhancing RUNX2 expression and alkaline phosphatase activity while also modulating Wnt/β-catenin signaling, a critical pathway for bone formation [11,58,59,60].

LYC modulates the RANKL/RANK/OPG pathway, a central regulator of bone remodeling, by influencing the expression of its key components and thereby shifting the balance toward bone preservation. Specifically, in an in vitro study using human osteoblast-like Saos-2 cells, treatment with LYC significantly reduced mRNA expression of RANKL, suggesting a downregulation of osteoclastogenic signaling pathways [11]. Similarly, LYC supplementation was able to improve the OPG/RANKL ratio in models of diabetic osteoporosis, as well as reducing inflammation and oxidative stress markers [41]. Mechanistically, this shift inhibits the interaction between RANKL and its receptor RANK on pre-osteoclasts, thereby suppressing osteoclast differentiation, activity, and survival, key processes in the pathogenesis of bone loss [61]. Moreover, LYC’s potent antioxidant capacity plays a complementary role by mitigating reactive oxygen species (ROS)-induced signaling pathways that promote osteoclastogenesis under oxidative stress conditions [11,41]. Together, these effects highlight the relevance of the RANKL/RANK/OPG axis in the observed bone-protective outcomes following LYC treatment.

Despite variability in LYC concentrations across studies, the findings collectively indicate a protective effect on bone health [11,17]. Several randomized controlled trials have demonstrated a significant reduction in bone resorption markers, such as CTX and NTx [35]. LYC supplementation, especially among postmenopausal women, has shown a modest increase in bone formation markers [34,48]. A 12-month clinical trial in postmenopausal women reported that LYC supplementation attenuated BMD loss at the lumbar spine [62]. Additionally, observational studies have associated higher dietary LYC intake with greater BMD in older adults [63].

This review has identified several key patterns in the literature. Notably, LYC appears to be effective in suppressing bone resorption, with postmenopausal women potentially benefiting more than other populations due to its protective effects against osteoporosis [33,48]. The beneficial effects were more evident at doses ≥15 mg/day [64,65,66], although the upper safe and effective limit has yet to be clearly defined. These findings suggest that LYC supplementation may be mainly advantageous for postmenopausal women at risk of osteoporosis, elderly individuals with osteopenia, and patients with chronic inflammatory conditions affecting bone health [11,35]. Furthermore, it is worth noting that co-supplementation with vitamin D and calcium may enhance the effects of LYC, as these nutrients contribute to improved mineral absorption and increased bone mineral density [67]. In addition, combining LYC with other phytonutrients has been shown to augment its antioxidant capacity [68].

In general, LYC was administered in a wide range of doses across both clinical and preclinical studies. In clinical trials, doses ranged from 8 to 150 mg/day, delivered through capsules or tomato-based products. These studies showed that LYC administration may decrease the risk of osteoporosis by decreasing oxidative stress and bone resorption, supporting cardiac and bone health, and preventing bone loss especially in postmenopausal women [11,33,35]. In animal studies, doses ranged from 10 to 100 mg/kg/day, with most studies using 10–50 mg/kg/day. Regarding the doses used in studies with animal models, higher concentrations of LYC were observed to produce pronounced biological effects. For instance, the dose of 50 mg/kg demonstrated greater efficacy compared to 10 mg/kg [30,43], while 40 mg/kg showed better results than 20 and 30 mg/kg [45]. Additionally, doses of 45 and 30 mg/kg were more effective than 15 mg/kg [17]. Similarly, the concentration of 100 ppm was found to be more effective than 50 ppm [44]. These findings suggest a possible dose-dependent relationship for the effects of LYC. In addition, LYC has been reported to distribute broadly into tissues, such as liver, adipose tissue, testes, adrenals, and skin, and it is metabolized through isomerization and β-oxidation, producing polar metabolites that are excreted in urine and carbon dioxide (CO_2_). The plasma level of LYC peaks after a single intake and then declines over the following days [69,70,71]. The plasma half-life of LYC is between 2–3 days, although some studies have reported longer values of approximately 12–33 days, depending on the methodology used [27,69]. In single-dose pharmacokinetic studies (10–120 mg doses), the elimination half-life of LYC was about 28 to 62 h (~1.2–2.6 days) and with a clearance (CL/F) between 98.6 and 286.4 mL/min. [70,71,72]. These pharmacokinetic features suggest that consistent, long-term intake may be necessary to achieve therapeutic effects [25]. However, the high variability in dosing and limited human data highlight the need for standardized clinical studies to determine optimal and effective doses for bone health.

Furthermore, it is important to highlight the findings of this review, specifically considering that, overall, the studies included presented a low risk of bias. Among the RCTs, only one study [35] failed to clearly report the allocation concealment process, representing a potential selection bias. In non-randomized clinical studies, a notable limitation was the short follow-up period for outcome assessment [37], which may weaken the strength of the conclusions. Nevertheless, the clinical trials generally exhibited adequate methodological quality in their conduct. Regarding the studies in animal models, certain methodological aspects remained unclear. For instance, the administration of LYC by gavage presents logistical challenges, and although not explicitly described in the studies, housing animals from experimental and control groups in separate cages may introduce unintentional environmental biases [73]. Without blinding, proper randomization becomes even more crucial to mitigate overestimation of treatment effects [74]. The omission of these procedures may lead to overestimation of treatment effects, whereas appropriate randomization and blinded outcome assessment strengthen the internal validity of the results. In addition, one study included in this review [40] was funded by industry sources, underscoring the importance of transparency in reporting to mitigate potential conflicts of interest. Finally, although a few studies have used small sample sizes, describing the sample calculation to justify the sample size is essential to ensure statistical power in the analysis of results.

Despite encouraging findings, this review has some limitations related to the current state of the literature. A meta-analysis was not conducted due to substantial heterogeneity among the included studies. This variability involved differences in study design, LYC dosage and formulation, duration of intervention, outcome measures (e.g., BMD, bone turnover markers), and target populations. Such clinical and methodological diversity made it inappropriate to statistically pool the data. As a result, a narrative synthesis was adopted to better capture and interpret the findings. The lack of standardized protocols and consistent outcome reporting further limited the feasibility of a quantitative approach, highlighting the need for greater methodological consistency in future research. Furthermore, the inclusion of clinical and pre-clinical studies provides a more comprehensive overview of the effects of LYC, combining the relevance of clinical research with the knowledge from pre-clinical studies. However, preclinical studies have challenges, such as differences in species, dosage, metabolism and controlled experimental conditions, that limit the direct translation of their results to humans. Despite these challenges, this mixed evidence approach helps to fill evidence gaps and guide future research. In addition, several studies have failed to demonstrate significant improvements in bone mineral density with LYC supplementation [75,76]. The optimal dosage remains unclear, and the limited number of available clinical trials is further constrained by small sample sizes and short follow-up periods, often less than one year. While LYC’s antioxidant and anti-inflammatory mechanisms are well-documented in animal models, further rigorous clinical investigations are needed to substantiate its efficacy and applications. Future research should focus on determining optimal dosing regimens, establishing long-term safety profiles, and exploring potential synergistic formulations. The existing data support the inclusion of LYC-rich foods in bone-healthy diets, with judicious supplementation offering additional benefits for at-risk populations. Thus, LYC should be considered a complementary strategy in the prevention and management of osteoporosis.

## 4. Materials and Methods

### 4.1. Protocol and Registration

This systematic review was conducted following the guidelines outlined in the Preferred Reporting Items for Systematic Reviews and Meta-Analyses (PRISMA) 2020 checklist [77]. The research protocol for this review was also registered in the International Prospective Register of Systematic Reviews (PROSPERO) database (CRD42023417346) and was also guided by recent publications [78,79].

### 4.2. PICO Question and Search Strategy

A specific research question was formulated based on the Population, Intervention, Comparison, and Outcome (PICO) framework, namely “Does LYC supplementation improve the quality and parameters of bone tissue?” The search strategy was structured using the PICO model, incorporating both controlled vocabulary (MeSH terms) and free-text keywords commonly found in article titles and abstracts. The components of the PICO acronym are as follows:

- Population (P): Adult patients (aged ≥20 years), with no restrictions on gender or profession, or animal models.
- Intervention (I): Lycopene supplementation.
- Comparison (C): Placebo or another control.
- Outcome (O): The primary outcome was bone mineral density (BMD) due to its clinical relevance as a standard indicator of bone strength and predictor of fracture risk. Secondary outcomes included bone turnover markers (e.g., osteocalcin, alkaline phosphatase, and CTX), bone microarchitecture assessed by microtomographic or histomorphometric analyses, and fracture incidence or other surrogate endpoints indicative of bone health.

Searches were conducted in PubMed, Scopus, Web of Science, Embase, and Cochrane Library. The search strategy was initially formulated for PubMed using a combination of MeSH terms and keywords related to LYC and bone health, and then appropriately adapted for the syntax and indexing of each database (Appendix A Table A1). To enhance coverage, a manual screening of the reference lists of all included studies and relevant reviews was performed to identify additional eligible publications. To find unpublished or ongoing studies, the ClinicalTrials.gov registry was searched, and gray literature was reviewed using the System for Information on Gray Literature in Europe (SIGLE) database. No date or language restrictions were applied. This systematic strategy aligns with PRISMA 2020 guidelines to ensure transparency and reproducibility. Two independent reviewers (ANAS and GPN) conducted the electronic search for studies published up to 8 May 2025. EndNote X8 software (Clarivate Analytics, Philadelphia, PA, USA) was used to organize the retrieved studies and citations.

### 4.3. Eligibility Criteria

Studies were included in this review if they met the following criteria: (a) studies involving adult participants aged 20 years or older, without restrictions on gender or occupation. Studies using animal models were also included; (b) studies assessing the effects of LYC supplementation compared to a placebo or no treatment group; (c) studies evaluating bone parameters as the primary outcome and/or other secondary outcomes; (d) studies focused on populations who had not received any treatment for bone disorders within 90 days prior to LYC supplementation and specimen collection.

Studies were excluded if they met any of the following conditions: (a) studies without a placebo or no treatment group; (b) studies that did not evaluate the outcomes of interest; (c) studies combining LYC supplementation with other treatments (e.g., anti-osteoporotic drugs); (d) clinical case reports and case series; (e) review articles.

### 4.4. Selection of Studies and Data Collection

Studies were selected based on their titles and abstracts, in accordance with the predefined eligibility criteria. Articles found in multiple databases were included only once. When the title and abstract did not provide sufficient information for eligibility assessment, the full-text article was reviewed. Two independent reviewers (ANAS and DVAPD) conducted the full-text screening and data extraction. Extracted data were subsequently tabulated and verified. In cases of disagreement, consensus was sought through discussion. When needed, a third reviewer (GPN) was consulted to resolve any remaining discrepancies [80].

The following variables were extracted from each study: authors/year, country of origin, study design, groups, number of subjects (n), sex, mean age, sample characteristics, administration of LYC protocol, evaluation methods, outcomes and results, conclusion, and effect of the intervention. Inter-examiner agreement during the selection process was calculated using the kappa score. Any disagreements were settled through discussion and consensus among all authors.

### 4.5. Quality Assessment and Risk of Bias of Individual Studies

The risk of bias for each study was assessed using the appropriate tool based on the study design. For randomized clinical trials (RCTs), the Cochrane Handbook for Systematic Reviews of Interventions (RoB 2.0) was used to evaluate the risk of bias, focusing on various domains, such as random sequence generation, allocation concealment, blinding, incomplete outcome data, and selective reporting. Each domain was rated as having a “high risk”, “low risk”, or “unclear risk” of bias (indicating insufficient information or uncertainty) [80].

Non-randomized studies, such as prospective studies, were evaluated using the Newcastle–Ottawa Scale (NOS). This tool evaluates three key components, namely selection, comparability, and outcomes. A study could earn up to 9 stars, with 6 or more stars indicating a low risk of bias, and 5 or fewer stars representing a high risk. Four stars were assigned for selection, two for comparability, and three for outcomes [81].

Studies in animal models were assessed using the SYRCLE’s RoB tool, with modifications as recommended by Hooijmans et al. (2014) [73]. Key factors evaluated included allocation sequence generation, baseline group similarity, allocation concealment, blinding, random sampling, handling of incomplete outcome data, and selective reporting. Additional considerations included sample size justification. Responses were categorized as “yes” (low risk of bias), “no” (high risk of bias), or “unclear” (insufficient information or uncertainty).

Two independent authors (ANAS and DVAPD) conducted the bias assessments. Any disagreements were resolved through discussion, and if consensus could not be reached, a third examiner (GPN) was consulted.

## 5. Conclusions

LYC shows promise as a natural supplement that may help protect bone health, especially in people at risk of osteoporosis and bone loss. However, current research is still limited, and most of the available data come from animal studies. To better understand its benefits, well-designed clinical trials are needed. Such studies are essential to generate robust evidence and support more confident clinical decision-making.

## Figures and Tables

**Table 1:** General characteristics of the clinical trials included in the review.

Author, Year (Location)	Study Design	Groups Number of Subjects (*n*); Mean Age	Characteristics of the Samples Included	Administration Protocol Lycopene	Assessment Method	Outcomes: Results	Intervention Effect	Conclusions
Meeta et al., 2022 [33] (India)	Multi-centric placebo-controlled double-blind randomized clinical trial	G1: Lyc; *n* = 60 G2: Placebo/*n* = 48 108 postmenopausal women (mean age—49.8 years)	Healthy postmenopausal women	LycoRed with a dosage of two capsules of 2 mg each was given twice daily after meals, 8 mg/day	ELISA; CLIA-COB S 411; liquid chromatography	Bone markers (ng/mL) Baseline/6 months G1: 0.5 ± 0.28/−0.13 ± 0.7 G2: 0.3 ± 0.07/−0.04 ± 0.1 P1NP (ng/mL) Baseline/6 months G1: 70.3 ± 11.45/−9.70 ± 25.0 G2: 50.5 ± 6.92/−2.96 ± 10.2	Positive	This study highlights the potential of lycopene supplementation in supporting cardiac and bone health.
Russo et al., 2020 [11] (Italy)	Clinical study	G1: (lycopene) *n* = 39 G2: (control) *n* = 39 The mean age of the enrolled population was 63 ± 7 years.	Postmenopausal women	Lycopene-rich tomato sauce daily, from tomatoes ripened on-the-vine at a dose of 150 mg/day	Real-time PCR; chemiluminescent immunoassay on COBAS 8000; immunoassay on Liaison^®^ XL; high-performance liquid chromatography.	G1/G2 BMD (g/cm^2^) Baseline: 0.39 ± 0.07; 0.41 ± 0.1 12 Weeks: 0.39 ± 0.08/0.38 ± 0.08 Bone alkaline phosphatase (ug/L) Baseline: 18.1 ± 7.0/19.2 ± 6.9 12 weeks: 14.6 ± 5.9/17.2 ± 6.7	Positive	Daily consumption of 150 mg of lycopene-rich tomato sauce for three months prevented bone loss in postmenopausal women.
Mackinnon et al., 2011 [34] (Canada)	Clinical study	G1: lycopene; 23 healthy postmenopausal women age (years) 54.4 ± 0.6	Postmenopausal women	Participants were given a list of lycopene-containing foods to avoid for the remainder of the study. Another set of dietary records and a fasting blood sample were collected following a one-month washout period.	High-performance liquid chromatography, Trolox-equivalent antioxidant capacity (TEAC) assay, ELISA	Lycopene restriction: one month Protein thiol: decreased from 423.7 mM ± 19.31 to 392.3 mM ± 14.22 TBARS: increase from 8.1 nmol/mL ± 0.4 to 9.18 nmol/mL ± 0.76; Protein oxidation: high 5.5% ± 3.3 Lipid peroxidation: high 14.5% ± 7.1; Endogenous antioxidant enzymes; CAT decrease was 8.4% ± 9.3; and SOD 22.7% ± 11.8 Bone resorption marker: NTx increase of 20.6% ± 9.8.	Positive	Lycopene-rich products in the daily diet may help maintain overall health and reduce the risk of age-related chronic diseases, especially osteoporosis.
Mackinnon et al., 2011 [35] (Canada)	Randomized controlled trial	G1.1: 15 mg lycopene tomato juice (30 mg/day), *n* = 15; age 55.2 ± 0.8; G1.2: 35 mg in the form of lycopene-rich tomato juice (70 mg/day). *n* = 15. Age 56.1 ± 0.64; G1.3: 15 mg lycopene in the form of tomato lycopene capsules (30 mg/day), *n* = 15, age: 54.3 ± 0.7; G2: placebo, *n* = 15, age: 55.1	Postmenopausal women	Following a 1-month washout without lycopene consumption, participants consumed either treatment (*n* = 15/group) twice daily for total lycopene intakes of 30, 70, 30, and 0 mg/day, respectively, for 4 months.	High-performance liquid chromatography, Trolox-equivalent antioxidant capacity (TEAC) assay, ELISA	Bone turnover markers BAP (U/L) G1.1: 22.3 ± 2.3; G1.2: 25.1 ± 1.98 G1.3: 23.1 ± 1.59; G2: 24.03 ± 2.36 Bone turnover markers-NTx (nM BCE): G1.1: 25.3 ± 2.16; G1.2: 22.64 ± 1.70; G1.3: 24.65 ± 2.12; G2: 20.25 ± 1.76	Positive	Lycopene intervention, given in capsule or juice form, supplying at least 30 mg/day, may decrease the risk of osteoporosis by decreasing oxidative stress and bone resorption.
Mackinnon et al., 2010 [36] (Canada)	Clinical study	107 female participants 25–70 years; genotypes 172T-A, 5 84A-G Age in years: 49.50	Blood samples	Using the USDA National Nutrient Database as a reference, lycopene content was calculated in milligrams for each food item, and the average daily intake was determined for each participant.	Blood genomic DNA isolation Kit; high-performance liquid chromatography; antioxidant capacity assay; ELISA	Genotype 172T→A NTx (nM BCE): TT 20.48 ± 0.9; TA 21.5 ± 1.3; AA 19.9 ± 2.1 TBARS (nmol/mL): TT 7.6 ± 0.4; TA 7.9 ± 0.4; AA 6.9 ± 0.5 BAP (U/L): TT 20.6 ± 1.2; TA 22.9 ± 1.1; AA 29.03 ± 2.4 Genotype 584A→G NTx, nM BCE: AA 20.9 ± 1.0; AG 21.39 ± 1.30; GG 17.78 ± 2.50 TBARS, nmol/mL: AA 7.93 ± 0.40; AG 7.36 ± 0.39; GG 7.11 ± 1.05 BAP (U/L): AA 23.98 ± 1.17; AG 21.26 ± 0.98; GG 21.71 ± 3.60	Positive	It suggests that in women with the 172TT genotype, a lycopene-rich diet is associated with lower bone resorption markers and may reduce the overall risk of osteoporosis.
Rão et al., 2007 [37] (Canada)	Clinical study	33 postmenopausal women (four groups, record diet G1; G2; G3; G4) The participants were grouped according to quartiles of serum lycopene per kg body weight (nM/kg). Age in years: 56.33 ± 0.45	Women between 50–60 years old who were at least one year postmenopausal	Participants were asked to sign an informed consent form, record their diet for seven days, and give a 12 h fasting blood sample on the eighth day.	ELISA; lipid peroxidation measurement; protein oxidation (thiols) measurement. High-performance liquid chromatographic analysis	Protein thiols (μM): G1: 504.2 ± 16.3; G2: 501.9 ± 25.; G3: 457.4 ± 50.1: G 4: 592.2 ± 31.1 TBARS (nmol/mL serum): G1: 7.4 ± 1.01; G2: 5.1 ± 0.30; G3: 5.04 ± 0.6; G4: 5.5 ± 0.59 NTx (nM BCE): G1: 22.5 ± 2.2; G2: 27.1 ± 2.3; G 3: 24.5 ± 1.4; G4: 17.1 ± 1.3; BAP (U/L): G1: 23.0 ± 2.7; G2: 20.7 ± 2.6; G3: 21.7 ± 3.3; G4: 21.3 ± 2.0	Positive	The lycopene in the participants’ daily diet appeared to be bioavailable and may help reduce bone resorption in postmenopausal women.

BAP: Bone alkaline phosphatase; BMD: bone mineral density; CAT: Catalase; CTx: C-terminal telopeptide of type I collagen; ELISA: enzyme-linked immunosorbent assay; Lyc: lycopene; NTx: N-telopeptide; P1NP: amino-terminal propeptide of type I collagen; Real-time PCR: real-time polymerase chain reaction; SOD: superoxide dismutase; TBARS: thiobarbituric acid reactive substance; BCE: bone collagen equivalents; TEAC: Trolox-equivalent antioxidant capacity.

**Table 2:** General characteristics of preclinical (animal models) studies included in the review.

Authors, Year (Location)	G1: Lycopene Use G2: Comparison Group Number of Subjects (*n*); Mean Age	Characteristics of the Samples Included	Administration Protocol Lycopene	Assessments Method	Outcomes: Results	Intervention Effect	Conclusion
Xia et al., 2024 [28] (China)	G1.1: LYC low-dose (LYCL) 15 mg/kg; G1.2: LYC high-dose (LYCH) 30 mg/kg; G2.1: SHAM with equal volume of sunflower oil; G2.2: ovariectomized the equal volume of sunflower oil; G2.3: estradiol valerate 0.1 mg/kg in sunflower oil; 10 per group; 230 ± 10 g, 11 weeks age	Ovariectomized and sham rats	(LYCH, 30 mg/kg; LYCL, 15 mg/kg) dissolved in sunflower oil, respectively (intragastric administration)	ELISA, Alizarin Red S staining, pathologic oil Red O staining, micro-CT, bone biomechanical strength assay, immunohistochemistry	G1.1/G1.2/G2.1/G2.2/G2.3 Trabecular thickness (mm): 1.93 ± 0.037; G1.2: 1.91 ± 0.033/1.90 ± 0.06/1.82 ± 0.03/1.9 ± 0.02 Bone mineral density (g/cm^3^): 0.13 ± 0.01/0.14 ± 0.01/0.12 ± 0.01/0.1 ± 0.01/0.12 ± 0.0 Bone volume (mm): G1: 38 ± 5; G1.2: 35 ± 1; G2: 40 ± 3.5; G2.1: 25 ± 2; G2.2: 37 ± 2.1	Positive	Lycopene may attenuate bone loss through the promotion of osteogenesis and inhibition of adipogenesis via the regulation of redox homeostasis in OVX rats.
Ricardo et al., 2023 [39] (Brazil)	G1: ovariectomized + LYC (OvxL) 45 mg/kg LYC) G2.1: ovariectomized (Ovx) 45 mg/kg water G2.2: surgery simulation: sham 15 female rats (5/group) (200 g)	Ovariectomized and sham rats	Lycopene 10% was diluted in water in the concentration of 45 mg/kg and administered daily by gavage the day after ovariectomy surgery for 16 weeks	Morphological and morphometrical analyses	G1/G2.1/G2.2 Neoformed bone area (mm^2^): 13.52 ± 3.38/5.62 ± 2.48/5.69 ± 3.61 Neoformed bone percentage: 26.36 ± 4.44/12.6 ± 2.49/16.69 ± 6.12	Positive	Lycopene at a concentration of 45 mg/kg stimulates bone repair, promoting significant bone formation in the absence of estrogenic hormone
Wang et al., 2023 [38] (China)	G1:SAMP6 + LYC (SAMP6 + LYC) 10 mg LYC dissolved in corn oil G2.1: SAMP 6 + Veh-gavage with corn oil (5 mL kg^−1^ day^−1^). G2.2: SAMR1 + Veh-gavaged with corn oil (5 mL kg^−1^ day^−1^) corn oil 30 male mice (10 per group)—3 months	Senile osteoporosis	Mice in the SAMP6 + LYC group were gavaged with lycopene (50 mg kg^−1^ day^−1^) dissolved in corn oil (10 mg lycopene mL^−1^) for 8 weeks.	Dual-energy X-ray absorptiometry, micro-CT, Serum biomarkers analysis, histology and histomorphometry, immunohistochemical analysis, Western blot assay, quantitative real-time PCR	G1/G2.1/G2.2 Trabecular thickness (um): 45 ± 1.41/42 ± 1.41/56 ± 1.41 Bone mineral density total (mg/cm^2^): 220 ± 14.14/150 ± 7.1/250 ± 7.1 Bone volume (%): 12 ± 1.1/7 ± 1.1/16 ± 2.1 Total bone mineral content (mg): 180 ± 14.4/120 ± 17.7; 125 ± 14.4	Positive	Demonstrate that the dietary intake of lycopene may provide a novel therapeutic strategy for the treatment of aging-related osteoporosis
Semeghini et al., 2022 [30] (Brazil)	G1: ovariectomized + LYC (OVX/Lyc)−1 mL of the solution containing 10 mg/kg of lycopene/*n* = 5 G2.1: (OVX)/the same volume of filtered water without lycopene/*n* = 5 G2.2: sham (control) the same volume of filtered water without lycopene//*n* = 5 Fifteen 2-month-old female rats	Ovariectomized and sham rats	Daily intragastric administration by oral gavage of 10 mg/kg body weight lycopene 10% was conducted for a period of 8 weeks	Micro-CT, quantitative gene expression—real-time PCR, stereological analysis	G1/G2.1/G2.2 Trabecular number (1/mm): 2.7 ± 0.1/G2: 2.3 ± 0.1/2.6 ± 0.1 Bone surface (mm^2^): 50 ± 3/130 ± 7/150 ± 7 Bone volume (mm^3^): 4.1 ± 0.1/3.7 ± 0.1/4.8 ± 0.1 Number of osteoblasts: 9000 ± 636/6000 ± 141.4/5500 ± 282 Number of osteoclasts: 1000 ± 35/3000 ± 70/5000 ± 141 Number of osteocytes: 60,000 ± 14,142.13/50,000 ± 1414/60,000 ± 707	Positive	Lycopene influences bone metabolism and may be a factor aiding in the prevention of bone loss occurring with the onset of osteoporosis
Xia et al., 2022 [29] (China)	G1: LYC 15 mg/kg G2.1: high-fat diet G2.2: metformin (500 mg/kg G2.3: normal control 9 per group; male mice (20 ± 2 g)	High-fat diet-induced Obese mice	Lycopene (15 mg/kg, dissolved in sunflower oil) for an additional 10 weeks.	Micro-CT, bone biomechanical strength and material profile assays, immunohistochemical analysis	G1/G2.1/G2.2/G2.3 Bone mineral density (g/cm^3^): 1.5 ± 0.07/1.5 ± 0.07/G2.1: 1.5 ± 0.07/1.5 ± 0.07 Bone surface density (mm): 9 ± 2.1/7 ± 1.4/7 ± 3.5/8 ± 2.1 Trabecular thickness (mm): 0.85 ± 0.38/0.8 ± 0.03/0.7 ± 0.49/0.8 ± 0.07 Cortical bone area (mm^2^): 1.1 ± 0.1/1.2 ± 0.1/1.3 ± 0.1/1.0 ± 0.1	Positive	Dietary supplementation of lycopene may offer a new therapeutic strategy for the management of obesity and its associated-osteoporosis
Mannino et al., 2022 [40] (Italy)	G1: (MP + LYCO) G2.1: (MP + Ale) G2.2: (MP + GEN) G2.3: (MP + GEN + LYCO) G2.4: SHAM G2.5: (MP) 10 per group; female Sprague–Dawley (SD) rats (*n* = 60), 5 months of age (250–275 g)	Osteoporosis glucocorticoid-induced osteoporosis rats	All treatments were daily administered per os through gavage and lasted an additional 60 days with lycopene 10 mg/kg days and associations	RT-qPCR, histology, micro-CT	G1/G2.1/G2.2/G2.3/G2.4/G2.5 Trabecular thickness (micron): 70 ± 5. 65/72 ± 2.12/62 ± 9.89/71 ± 8.42/78 ± 2.82/60 ± 2.12 Bone mineral density (mg/cm^3^): 620 ± 63.6/700 ± 35.3/600 ± 77.7/650 ± 98/780 ± 84/430 ± 28 Bone volume (BV) (Trabecular): 28% ± 1/30% ± 3/25% ± 3/28%± 5/30% ± 2/22% ± 2 Bone volume (BV) Cortical: 64% ± 4.2/62% ± 5.6/58% ± 7.1/64% ± 5/75% ± 5.64/50% ± 5	Positive	Combined treatment of genistein and lycopene significantly restored normal architecture and adequate interconnectivity between the bone trabeculae, thus increasing BMD levels
Qi et al., 2021 [41] (China)	G1.1: (LYC-L) G1.2: (LYC-H) G2.1: (control) G2.2: (diabetic) G2.2: (metformin) Female rats, 8 weeks old 12 per group; (210 g~223 g)	Diabetic rats	G1.1: (LYC-L): treated with lycopene 50 mg/kg/day G1.2: (LYC-H): treated with lycopene 100 mg/kg/day oral gavage for 10 weeks	Micro-CT, bone mechanical parameters measurement, ELISA, bone histomorphometry	G1.1/G1.2/G2.1/G2.2 Trabecular thickness (mm) 0.8 ± 0.1/0.9 ± 0.5/0.1 ± 0.01/0.6 ± 0.1/0.9 ± 0.49 Bone volume (mm^3^): 5.30 ± 0.14/5.8 ± 0.14/7.5 ± 0.70/3.0 ± 0.14/6.5 ± 0.07 Bone surface area (mm^2^) 170 ± 14.14/200 ± 7.07/230 ± 10.60/110 ± 3.53/180 ± 14.14 Trabecular volume (mm^3^): 32 ± 2.1; 33 ± 2.1/41 ± 0.1/22 ± 1.1/34 ± 2.8	Positive	Lycopene could prevent diabetic induced bone loss via anti-inflammatory and antioxidant effects, inhibiting bone resorption, upregulating OPG/RANKL ratio, and regulating abnormal bone turnover
Oliveira et al., 2019 [32] (Brazil)	G1.1: sham + daily intake of 10 mg/kg of LYC for 30 day G1.2: sham + daily intake of 10 mg/kg of LYC for 60 days G1.3: OVX + daily intake of 10 mg/kg of LYC for 30 day G1.4: OVX + daily intake of 10 mg/kg of LYC for 60 days G2.1: sham group G2.2: OVX 3 per group, Wistar female rats weighing approximately 300 g	Ovariectomized and sham rats osteoporosis	Lycopene (10 mg/kg weight per day) dissolved in filtrated water by daily intragastric administration for experimental periods of 30 and 60 days. The group that received 30 days of lycopene had the administration substituted for filtrated water for the other 30 days until killing	Alizarin Red S, real-time PCR, In situ alkaline phosphatase assay, alkaline phosphatase activity, mineralized matrix formation and real-time PCR, histomorphometry	G1.1/G1.2/G1.3/G1.4/G2.1/G2.2 Trabecular bone (%): 30 ± 2.12/32 ± 1.41/23 ± 0.70/30 ± 0.70; 35 ± 0.70/12 ± 1.41	Positive	Daily intake of lycopene for 30 or 60 days decreased bone loss in femur epiphysis. Thus, lycopene might be a potential adjuvant to drug therapy used in the prevention and treatment of osteoporosis
Mityas et al., 2019 [42] (Egypt)	G1.1: LYC 30 mg/kg G1.2 LYC + prednisolone G2.1: (control) G2.2: prednisolone 10 per group; adult male rats 150–200 g	Glucocorticoid-induced osteoporosis rats	G1: lycopene orally 30 mg/kg once daily for 8 weeks; G2: lycopene orally at a dose of 30 mg/kg BW once daily and prednisolone orally at a dose of 20 mg/kg BW once daily for 8 weeks	Hematoxylin and eosin stain; Mallory’s trichrome stain, C-scanning electron microscopy	G1.1/G1.2/G2.1/G2.2 Area percentage—collagen fiber contents: 32.4 ± 4.6/30.0 ± 4.5/31.2 ± 3.5/16.5 ± 4.1	Positive	It is recommended that lycopene can be used as a dietary alternative to drug therapy or as a supplement to people at risk for osteoporosis
Li et al., 2018 [43] (China)	G1: OVX + LYC G2.1: OVX G2.2: SHAM Thirty female (ten per group) Sprague–Dawley rats aged 12 weeks old with a weight of 245 ± 7.46 g	Ovariectomized rats	Lycopene (50 mg/kg/day)	Biomechanical tests, micro-CT, histological analysis	G1/G2.1/G2.2 Bone mineral density(mg/cc): 160 ± 17.8/140 ± 14.1/170 ± 14.1 Trabecular thickness (μm): 125 ± 7.07/100 ± 17.67/130 ± 9.19 Bone volume (%): 25 ± 2.1/18 ± 1.4/24 ± 4.2 Trabecular separation: 560 ± 7/600 ± 14/530 ± 17 Trabecular number: 1.6 ± 0.2/1.5 ± 0.1/1.7 ± 0.1	Positive	Lycopene significantly increased implant osseointegration, fixation, and bone mass in OVX rats to the level of those in sham rats
Ardawi et al., 2016 [17] (Saudi Arabia)	G1.1: LYC-supplemented (15 mg/kg weight per day) G1.2: LYC-supplemented (30 mg/kg weight per day); G1.3 OVX lycopene-supplemented (45 mg/kg per day); G2.1: OVX alendronate-treated (ALN) [2.0 μg/kg body weight per day subcutaneously; G2.2: sham-operated; G2.3: ovariectomized control 44 animals per group Six-month-old female rats (*n* = 264)	Ovariectomized and sham rats	The lycopene-supplemented groups were given lycopene (15, 30, and 45 mg/kg body weight per day) dissolved in corn oil by daily intragastric administration for the experimental period of 12 weeks. The SHAM, OVX, and ALN control groups were given the same volume of corn oil without lycopene treatment	858 Mini Bionex Servohydraulic Test System; micro-CT	G1.1/G1.2/G1.3/G2.1/G2.2/G2.3 μCT: relative bone volume (%) 17.6 ± 1.6/26.7 ± 1.9/31.6 ± 1.8/32.3 ± 1.9/31.0 ± 1.4/14.1 ± 0.3 Trabecular number (mm): 3.9 ± 0.1/4.8 ± 0.2/5.2 ± 0.1/5.1 ± 0.1/5.1 ± 0.1/3.4 ± 0.1 Trabecular thickness (mm): 0.07 ± 0.0/0.07 ± 0.01/0.09 ± 0.01/0.08 ± 0.01/0.08 ± 0.01/0.07 ± 0.01; Femur diaphysis—relative bone volume (%): 62.04 ± 0.60/65.00 ± 0.49/66.77 ± 0.99/67.45 ± 0.73/65.19 ± 0.67/61.88 ± 0.67 Cortical thickness (mm): 0.624 ± 0.011/0.69 ± 0.012/0.7 ± 0.01/0.7 ± 0.01/0.65 ± 0.01/0.615 ± 0.01 Cortical porosity (%): 0.180 ± 0.01/0.21 ± 0.00/0.22 ± 0.01/0.23 ± 0.01/0.21 ± 0.01/0.28 ± 0.01	Positive	Lycopene treatment for 12 weeks demonstrated bone-protective effects similar to ALN, improving the biomechanical properties of bone and inhibiting bone resorption in OVX rats
Iimura et al., 2015 [18] (Japan)	G1.1: LYC 50 mg/kg, *n* = 9 G1.2: LYC 100 mg/kg, *n* = 9 G1.3: LYC 200 mg/kg, *n* = 9 G2: lycopene 0 mg/kg, *n* = 6 Female 6-week-old rats (*n* = 33)	Ovariectomized rats	Based on the lycopene content in their diet (0, 50, 100, and 200 ppm (mg lycopene/kg diet)); lycopene was incorporated into the diet as a tomato extract at 6%	HPLC analysis.; dual-energy X-ray absorptiometry; ELISA, d-ROMs test, biological antioxidant potential (BAP) test	G1.1/G1.2/G1.3/G2 Serum bone-type ALP (mU): 56.1 ± 4/56 ± 4.7/47 ± 3.6/59 ± 3.4 Serum d-ROMs level (U. CARR): 224 ± 15/235 ± 9/216 ± 11/239 ± 8 Serum BAP (l mol): 2738 ± 81/2741 ± 61/2851 ± 63/±75 Serum BAP/d-ROMs ratio: 12.2 ± 0.6/11.8 ± 0.5/13.4 ± 0.4/11.9 ± 0.6 8-OHdG excretion (lg/day): 1.2 ± 0.1/1.3 ± 0.3/1.5 ± 0.3/1.0 ± 0.2	Positive	Lycopene intake significantly inhibited bone resorption, thereby suppressing bone loss in ovariectomized rats, but failed to alter the systemic oxidative stress level
Iimura et al., 2014 [44] (Japan)	G1.1: LYC 50 mg/kg; G1.2: LYC 100 mg/kg; G2: LYC 0 mg/kg; 8 per group; Six-week-old female rats	Growing female rats	According to the lycopene content in their diet: 0, 50, and 100 ppm. Lycopene was incorporated into the diet as a tomato extract containing 6% lycopene	Dual-energy X-ray absorptiometry, femoral mechanical braking test, urinary deoxypyridinoline, serum bone type ALP activity	G1.1/G1.2/G2 Femoral breaking force: 21.19 ± 0.40/21.05 ± 0.29/20.41 ± 0.48 Femoral breaking energy: 12.8 ± 0.88/13.8 ± 1.24/12.1 ± 1.1 Bone turnover markers (nmol/mg): 0.19 ± 0.03/0.2 ± 0.03/0.34 ± 0.5 Serum ALP: 44.9 ± 4.6/48.6 ± 4.1/35.7 ± 2.1	Positive	Lycopene intake facilitated bone formation and inhibited bone resorption, which contributed to an increase in the BMD of growing female rats
Liang et al., 2012 [45] (China)	G1.1: OVX lycopene 20 mg/kg body weight G1.2: OVX lycopene 30 mg/kg G1.3: OVX lycopene 40 mg/kg G2.1: control group (OVX) G2.2: placebo group *n* = 50–2-month-old female rats (body weight 225 ± 10 g)	Ovariectomized mature rats	Lycopene 20, 30, and 40 mg/(kg body weight day) dissolved in corn oil, respectively, by intragastric administration for 8 weeks	X-ray absorptiometry; analysis of serum Ca, P concentration and serum ALP, IL-6, estrogen, BGP, ELISA; three-point bending test	G1.1/G1.2/G1.3/G2.1/G2.2 Serum Ca, P, and ALP Ca (mmol/L): 2.55 ± 0.22/G1.2: 2.52 ± 0.21/2.45 ± 0.25/2.4 ± 0.17/2.61± 0.19 P (mmol/L): 1.56 ± 0.18/1.5 ± 0.16/1.4 ± 0.2/1.3 ± 0.2/: 1.6 ± 0.2 ALP (U/L): 83.4 ± 7.32/79.22 ± 6.83/71.33 ± 8.28/60.5 ± 4.8/98.3 ± 6.9	Positive	Lycopene treatment can inhibit bone loss and increase bone strength in OVX rats
Pei et al., 2008 [46] (China)	G1.1: LYC: 10 mg/(kg) G1.2: LYC 15 mg/(kg) G1.3: LYC 20 mg/(kg) G2.1: Nylestriol (estrogen) G2.2: white group G2.3: control *n* = 72, 15-week-old SD rats	Ovariectomized rats	The nialestradiol group was given 1.05 mg/(kg-bw) by gavage, and the high, medium, and low doses of lycopene were given at 20, 15, and 10 mg/(kg-bw) by gavage. 12 weeks. The blank group and the model group received water	Chromatography, serum estradiol, serum ALP and uterus index, length bone mineral density, bone mineral	G1.1/G1.2/G1.3/G2.1/G2.2/G2.3 Bone mineral content (mg): 101.3 ± 9.0/104.5 ± 1/107.1 ± 10/112. 6 ± 6/98.13 ± 9.1/87.4 ± 7.0 Bone density BMC mg): 65.8 ± 5.0/67.2 ± 5.1/69.3 ± 5.0/78.6 ± 5.8/66.6 ± 4.4/55.2 ± 4.0	Positive	In short, lycopene has an estrogen-like effect. Effective in improving the occurrence of osteoporosis caused by postmenopause

8-OHdG: 8-hydroxy-2-deoxyguanosine; ALN: alendronate; ALP: alkaline phosphatase; BGP: bone gla protein; BMC: bone mineral content; BMD: bone mineral density; Dpd: daily urinary deoxypyridinoline; d-ROM: reactive oxygen metabolites-derived compounds; ELISA: enzyme-linked immunosorbent assay; HPLC: high-performance liquid chromatography; IL-6: Interleukin-6; LYC: lycopene; MET: metformin; MP: methylprednisolone; NC: normal control; Ovx: ovariectomized; SAMP6: senescence-accelerated mouse strain 6; SAMR1: senescence-accelerated mouse resistant 1; SHAM: sham-operated; TRAP: tartrate-resistant acid phosphatase; PCR: polymerase chain reaction; LYCO: lycopene; GEN: genistein; OPG/RANKL: Osteoprotegerin/Receptor Activator of Nuclear factor-Kappa B ligand; Micro-CT: microcomputed tomography; BAP: biological antioxidant potential.

**Table 3:** Risk of bias assessment of the selected non-randomized clinical studies using the Newcastle–Ottawa Scale.

Studies	Selection				Comparability		Outcome			Total
	Exposed Cohort	Non Exposed Cohort	Ascertainment of Exposure	Outcome of Interest Not Present at Start	Main Factor	Additional Factor	Assessment of Outcome	Follow-Up Long Enough	Adequacy of Follow-Up	
Russo et al., 2020 [11]	✰	✰	✰	✰	✰	✰	✰	✰	✰	8
Mackinnon et al., 2011 [34]	✰	✰	✰	✰	✰	0	✰	✰	✰	8
Mackinnon et al., 2010 [36]	✰	✰	✰	✰	✰	0	✰	✰	✰	8
Rão et al., 2007 [37]	✰	✰	✰	✰	✰	0	✰	0	✰	7

## Data Availability

No new data were created or analyzed in this study. Data sharing is not applicable.

## Appendix A

**Table A1:** Search strategy in the databases.

Search Strategy
PUBMED/MEDLINE ((((((((Bone[MeSH Terms]) OR ((Bone) OR (Bones and Bone Tissue) OR (Bones and Bone) OR (Bone and Bone) OR(Bone Tissue) OR (Bone Tissues) OR (Tissue, Bone) OR (Tissues, Bone) OR (Bony Apophyses) OR (Apophyses, Bony) OR (Bony Apophysis) OR (Apophysis, Bony) OR (Condyle) OR (Condyles) OR (Bones))) OR ((Bone Remodeling)[MeSH Terms])) OR ((Bone Remodeling) OR (Remodeling, Bone) OR (Bone Turnover) OR (Bone Turnovers) OR (Turnover, Bone) OR (Turnovers, Bone))) OR ((Osteogenesis)[MeSH Terms])) OR ((Osteogenesis) OR (Bone Formation) OR (Ossification) OR (Ossifications) OR (Osteoclastogenesis) OR (Osteoclastogeneses) OR (Endochondral Ossification) OR (Endochondral Ossifications) OR(Ossification, Endochondral) OR (Ossifications, Endochondral) OR (Physiologic Ossification) OR (Ossification, Physiological) OR (Physiological Ossification) OR (Ossification, Physiologic))) OR ((Bone Resorption)[MeSH Terms])) OR ((Bone Resorption) OR (Bone Resorptions) OR (Resorption, Bone) OR (Resorptions, Bone) OR (Osteoclastic Bone Loss) OR (Bone Loss, Osteoclastic) OR (Bone Losses, Osteoclastic) OR (Loss, Osteoclastic Bone) OR (Losses, Osteoclastic Bone) OR (Osteoclastic Bone Losses))) AND (((Lycopene[MeSH Terms])) OR ((Lycopene) OR (LYC-O-MATO) OR (LYC O MATO) OR (LYCOMATO) OR (All-trans-Lycopene) OR (All trans Lycopene) OR (Lycopene, (7-cis,7′-cis,9-cis,9′-cis)-isomer -) OR (Pro-Lycopene) OR (Pro Lycopene) OR (Prolycopene) OR (Lycopene, (cis)-isomer) OR (Lycopene, (13-cis)-isomer))) (http://www.ncbi.nlm.nih.gov/pubmed (accessed on 8 May 2025))
SCOPUS (TITLE-ABS-KEY ((lycopene) OR (lyc-o-mato) OR (lyc AND o AND mato) OR (lycomato) OR (all-trans-lycopene) OR (all AND trans AND lycopene) OR (lycopene) OR (pro-lycopene) OR (pro AND lycopene)) AND TITLE-ABS-KEY ((bone) OR (bones AND bone AND tissue) OR (bones AND bone) OR (bone AND bone) OR (bone AND tissue) OR (bone AND tissues) OR (tissue, AND bone) OR (tissues, AND bone) OR (bony AND apophyses) OR (apophyses, AND bony) OR (bony AND apophysis) OR (apophysis, AND bony) OR (condyle) OR (condyles) OR (bones) OR (bone) OR (bone AND remodeling) OR (remodeling, AND bone) OR (bone AND turnover) OR (bone AND turnovers) OR (turnover, AND bone) OR (turnovers, AND bone) OR (bone AND formation) OR (bone AND formation) OR (ossification) OR (ossifications) OR (osteoclastogenesis) OR (osteoclastogeneses) OR (endochondral AND ossification) OR (endochondral AND ossifications) OR (ossification, AND endochondral) OR (ossifications, AND endochondral) OR (physiologic AND ossification) OR (ossification, AND physiological) OR (physiological AND ossification) OR (ossification, AND physiologic) OR (bone AND resorption) OR (bone AND resorptions) OR (resorption, AND bone) OR (resorptions, AND bone) OR (osteoclastic AND bone AND loss) OR (bone AND loss, AND osteoclastic) OR (bone AND losses, AND osteoclastic) OR (loss, AND osteoclastic AND bone) OR (losses, AND osteoclastic AND bone) OR (osteoclastic AND bone AND losses))) (http://www.scopus.com (accessed on 8 May 2025))
EMBASE (‘lyc o mato’/exp OR ‘lyc o mato’ OR (lyc AND o AND (‘mato’/exp OR mato)) OR lycomato OR ‘all trans lycopene’ OR (all AND trans AND (‘lycopene’/exp OR lycopene)) OR ‘lycopene’/exp OR lycopene OR ‘pro lycopene’ OR (pro AND (‘lycopene’/exp OR lycopene))) AND (bones:ti,ab,kw AND ‘bone tissue’:ti,ab,kw OR (bones:ti,ab,kw AND bone:ti,ab,kw) OR ‘bone tissue’:ti,ab,kw OR ‘bone tissues’:ti,ab,kw OR ‘tissue, bone’:ti,ab,kw OR ‘tissues, bone’:ti,ab,kw OR ‘bony apophyses’:ti,ab,kw OR ‘apophyses, bony’:ti,ab,kw OR ‘bony apophysis’:ti,ab,kw OR ‘apophysis, bony’:ti,ab,kw OR condyle:ti,ab,kw OR condyles:ti,ab,kw OR bones:ti,ab,kw OR bone:ti,ab,kw OR ‘bone remodeling’:ti,ab,kw OR ‘remodeling, bone’:ti,ab,kw OR ‘bone turnover’:ti,ab,kw OR ‘bone turnovers’:ti,ab,kw OR ‘turnover, bone’:ti,ab,kw OR ‘turnovers, bone’:ti,ab,kw OR ‘bone formation’:ti,ab,kw OR ossification:ti,ab,kw OR ossifications:ti,ab,kw OR osteoclastogenesis:ti,ab,kw OR osteoclastogeneses:ti,ab,kw OR ‘endochondral ossification’:ti,ab,kw OR ‘endochondral ossifications’:ti,ab,kw OR ‘ossification, endochondral’:ti,ab,kw OR ‘ossifications, endochondral’:ti,ab,kw OR ‘physiologic ossification’:ti,ab,kw OR ‘ossification, physiological’:ti,ab,kw OR ‘physiological ossification’:ti,ab,kw OR ‘ossification, physiologic’:ti,ab,kw OR ‘bone resorption’:ti,ab,kw OR ‘bone resorptions’:ti,ab,kw OR ‘resorption, bone’:ti,ab,kw OR ‘resorptions, bone’:ti,ab,kw OR ‘osteoclastic bone loss’:ti,ab,kw OR ‘bone loss, osteoclastic’:ti,ab,kw OR ‘bone losses, osteoclastic’:ti,ab,kw OR ‘loss, osteoclastic bone’:ti,ab,kw OR ‘losses, osteoclastic bone’:ti,ab,kw OR ‘osteoclastic bone losses’:ti,ab,kw) AND [embase]/lim (https://www.embase.com (accessed on 8 May 2025))
WEB OF SCIENCE ((((((((Bone) OR ((Bone) OR (Bones and Bone Tissue) OR (Bones and Bone) OR (Bone and Bone) OR(Bone Tissue) OR (Bone Tissues) OR (Tissue, Bone) OR (Tissues, Bone) OR (Bony Apophyses) OR (Apophyses, Bony) OR (Bony Apophysis) OR (Apophysis, Bony) OR (Condyle) OR (Condyles) OR (Bones))) OR ((Bone Remodeling) OR ((Bone Remodeling) OR (Remodeling, Bone) OR (Bone Turnover) OR (Bone Turnovers) OR (Turnover, Bone) OR (Turnovers, Bone))) OR ((Osteogenesis) OR ((Osteogenesis) OR (Bone Formation) OR (Ossification) OR (Ossifications) OR (Osteoclastogenesis) OR (Osteoclastogeneses) OR (Endochondral Ossification) OR (Endochondral Ossifications) OR(Ossification, Endochondral) OR (Ossifications, Endochondral) OR (Physiologic Ossification) OR (Ossification, Physiological) OR (Physiological Ossification) OR (Ossification, Physiologic))) OR ((Bone Resorption) OR ((Bone Resorption) OR (Bone Resorptions) OR (Resorption, Bone) OR (Resorptions, Bone) OR (Osteoclastic Bone Loss) OR (Bone Loss, Osteoclastic) OR (Bone Losses, Osteoclastic) OR (Loss, Osteoclastic Bone) OR (Losses, Osteoclastic Bone) OR (Osteoclastic Bone Losses))) AND (((Lycopene) OR ((Lycopene) OR (LYC-O-MATO) OR (LYC O MATO) OR (LYCOMATO) OR (All-trans-Lycopene) OR (All trans Lycopene) OR (Lycopene, (7-cis,7′-cis,9-cis,9′-cis)-isomer -) OR (Pro-Lycopene) OR (Pro Lycopene) OR (Prolycopene) OR (Lycopene, (cis)-isomer) OR (Lycopene, (13-cis)-isomer))) (https://clarivate.com/webofsciencegroup/solutions/web-of-science-core-collection (accessed on 8 May 2025))
COCHRANE LIBRARY (Bone) OR (Bone) OR (Bones and Bone Tissue) OR (Bones and Bone) OR (Bone and Bone) OR (Bone Tissue) OR (Bone Tissues) OR (Tissue, Bone) OR (Tissues, Bone) OR (Bony Apophyses) OR (Apophyses, Bony) OR (Bony Apophysis) OR (Apophysis, Bony) OR (Condyle) OR (Condyles) OR (Bones) OR (Bone Remodeling) OR (Bone Remodeling) OR (Remodeling, Bone) OR (Bone Turnover) OR (Bone Turnovers) OR (Turnover, Bone) OR (Turnovers, Bone) OR (Osteogenesis) OR (Osteogenesis) OR (Bone Formation) OR (Ossification) OR (Ossifications) OR (Osteoclastogenesis) OR (Osteoclastogeneses) OR (Endochondral Ossification) OR (Endochondral Ossifications) OR(Ossification, Endochondral) OR (Ossifications, Endochondral) OR (Physiologic Ossification) OR (Ossification, Physiological) OR (Physiological Ossification) OR (Ossification, Physiologic) OR (Bone Resorption) OR (Bone Resorption) OR (Bone Resorptions) OR (Resorption, Bone) OR (Resorptions, Bone) OR (Osteoclastic Bone Loss) OR (Bone Loss, Osteoclastic) OR (Bone Losses, Osteoclastic) OR (Loss, Osteoclastic Bone) OR (Losses, Osteoclastic Bone) OR (Osteoclastic Bone Losses) in Title Abstract Keyword AND (Lycopene) OR (Lycopene) OR (LYC-O-MATO) OR (LYC O MATO) OR (LYCOMATO) OR (All-trans-Lycopene) OR (All trans Lycopene) OR (Lycopene, (7-cis,7′-cis,9-cis,9′-cis)-isomer -) OR (Pro-Lycopene) OR (Pro Lycopene) OR (Prolycopene) OR (Lycopene, (cis)-isomer) OR (Lycopene, (13-cis)-isomer) (Lycopene) OR (LYC-O-MATO) OR (LYC O MATO) OR (LYCOMATO) OR (All-trans-Lycopene) OR (All trans Lycopene) in Title Abstract Keyword (https://www.cochranelibrary.com (accessed on 8 May 2025))
